# Potential Therapeutic Role of L-Carnitine in Skeletal Muscle Oxidative Stress and Atrophy Conditions

**DOI:** 10.1155/2015/646171

**Published:** 2015-03-08

**Authors:** Anna Montesano, Pamela Senesi, Livio Luzi, Stefano Benedini, Ileana Terruzzi

**Affiliations:** ^1^Department of Biomedical Sciences for Health, University of Milan, Milan, Italy; ^2^Metabolism Research Center, San Donato Hospital and Scientific Institute, Milan, Italy; ^3^Diabetes Research Institute, Metabolism, Nutrigenomics and Cellular Differentiation Unit, San Raffaele Scientific Institute, 60 Olgettina street, 20132 Milan, Italy

## Abstract

The targeting of nutraceutical treatment to skeletal muscle damage is an emerging area of research, driven by the need for new therapies for a range of muscle-associated diseases. L-Carnitine (CARN) is an essential nutrient and plays a key role in mitochondrial *β*-oxidation and in the ubiquitin-proteasome system regulation. As a dietary supplement to improve athletic performance, CARN has been studied for its potential to enhance *β*-oxidation. However, CARN effects on myogenesis, mitochondrial activity, and hypertrophy process are not completely elucidated. This *in vitro* study aims to investigate CARN role on skeletal muscle remodeling, differentiation process, and myotubes formation. We analyzed muscle differentiation and morphological features in C2C12 myoblasts exposed to 5 mM CARN. Our results showed that CARN was able to accelerate C2C12 myotubes formation and induce morphological changes, characterizing the start of hypertrophy process. In addition, CARN improved AKT activation and downstream cellular signaling pathways involved in skeletal muscle atrophy process prevention. Also, CARN positively regulated the pathways involved in oxidative stress defense. In this work, we provide an interesting novel mechanism of the potential therapeutic use of CARN to treat pathological conditions characterized by skeletal muscle morphological and functional impairment, oxidative stress production, and atrophy process in aging.

## 1. Introduction

The role of many nutrients in maintaining good health and prolonging human lifespan has been clearly demonstrated over the past three decades. In particular, plant food stuffs, animal foods, and lipids have been shown to have protective effects against several chronic pathologies such as age-related diseases, including cardiovascular [1], neurodegenerative [2], and inflammatory diseases [3], diabetes [4], and myopathies [5]. In these pathologies, the skeletal muscle is the critical target.

The deterioration of skeletal muscle structure and function leads to clinically relevant complaints, including progressive strength loss, fatigue, myalgia, and cramps. Important progress has been made in the comprehension of the molecular mechanisms underlying muscle myopathies. However, the treatment of muscle diseases is mainly symptom-oriented and includes physical therapy and exercise, but no specific pharmacologic interventions are currently available [5, 6].

Considering the lack of therapies for sarcopenia and muscle atrophy, the idea that nutritional supplements might have beneficial effects in muscle damages treatment is experiencing renewed interest. Conclusions about how beneficial nutritional supplements are for myopathy treatment are complicated by a lack of unequivocal results and flaws in the choice of supplements. Based on the physiological roles in muscle biochemistry and bioenergetics, it is not surprising that carnitine role has been studied intensively.

Carnitine (CARN) is a derivative amino acid playing an essential role in cellular energy metabolism due to the acylation of its *β*-hydroxyl group and in the long-chain fatty acids transport into the mitochondrial matrix [7], where they undergo *β*-oxidation.

CARN can be supplied with diet, especially with foods of animal origin. The skeletal muscle is the most relevant depository of CARN (95% of the total body stores), and its availability is critical for the physiological bioenergetics of this tissue. CARN deficiency greatly affects skeletal muscle function as found in the presence of primary and secondary deficiencies [8].

In accordance with CARN role in skeletal muscle, a large amount of research was directed towards investigating the effects of CARN supplementation on exercise performance [9], particularly in ameliorating and accelerating recovery from exercise-induced muscle injury [7]. It has been found that supplemental CARN is effective in attenuating signs of tissue damage induced by lengthening or intense contractions also in sarcopenic muscle [10]. In particular, evidence from both animal and clinical studies demonstrated that CARN treatment positively influences many different mechanisms involved in pathologic skeletal muscle loss [11].

The observed benefits of CARN in preventing load-induced muscle injury have been attributed to its role as antioxidant [12]. In skeletal muscle, reactive oxygen species (ROS) and nitrogen species are physiologically synthesized at low levels and are required for normal force production. When ROS production overtakes tissue antioxidant capacity, oxidative stress activates pathophysiologic signaling leading to proteolysis and apoptosis within the myofibers [13]. This sequence of events is considered a major cause of sarcolemmal damage and the origin of reduced muscle strength capacity that contributes to fatigue [14].

However, the effects of CARN on myogenesis, myotubes formation, muscle atrophy, and muscle regeneration have not yet been completely elucidated.

Aim of this study is to clarify CARN effects on myogenesis in order to speculate a novel nutraceutical approach in the treatment of muscle injury and muscle atrophy process.

## 2. Materials and Methods

### 2.1. Materials

Mouse C2C12 myoblastic cells were purchased from the European Collection of Animal Cell Cultures (ECACC). Reagents were purchased from Sigma Chemical Co. (St. Louis, MO, USA). Primary antibodies against AKT (C-20), calnexin (H-70), CAMKII (M-176), ERK1 (K-23), ERK2 (C-14), GAPDH (FL-335), IGF-1 receptor *β* (C-20), Myf5 (c-20), MyHC (H-300), MyoD (C-20), myogenin (D-10), pERK1/2 (E-4), anti-p53 (FL-393), p70S6 (C-18), pp70S6 (sc-7984), SOD2 (FL-222), peroxidase-conjugated secondary antibodies for Western blot analysis and rhodamine-conjugated antibodies for Immunofluorescence analysis were purchased from Santa Cruz Biotechnology (Santa Cruz, CA, USA). Primary antibodies phospho-AKT (Ser473) (D9E) XP and phospho-AMPK alpha (Thr172) (40H9) were purchased from Cell Signaling Technology (Danvers, MA, USA). Antibody against Phalloidin (Alexa Fluor 488 Phalloidin, molecular probes-Invitrogen) was purchased by Life Technologies (Carlsbad, California, USA).

### 2.2. Cell Culture

C2C12 cells were maintained at 37°C in humidified 5% CO_2_ atmosphere in a growth medium (GM) containing DMEM (Dulbecco Modified Eagle Medium) supplemented with 20% (v/v) FBS (Fetal Bovine Serum), 1% penicillin streptomycin, and 1% L-glutamine up to 70% confluence. Cell differentiation was initiated by placing 70% confluent cell cultures in differentiation medium (DM), containing DMEM supplemented with 1% HS (horse serum), antibiotics, and 1% L-glutamine. In our* in vitro* differentiation model, early myotubes appeared 24–48 hours (h) after serum starvation and neomyotubes formation was completed after 72 h [15].

### 2.3. Experimental Procedures

**Figure 1:**
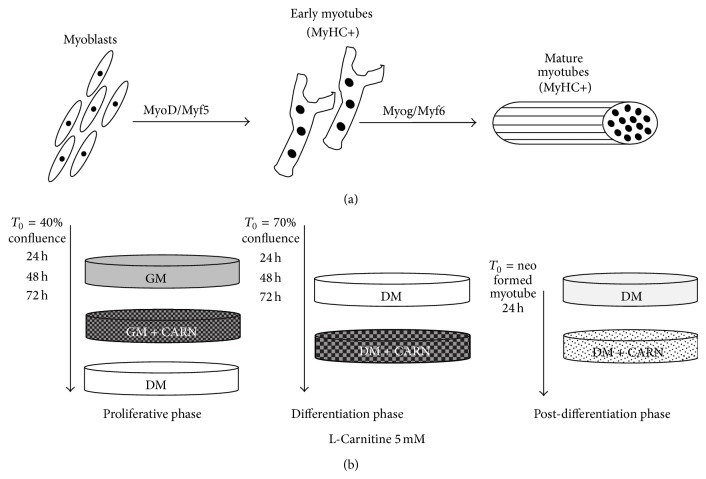
Experimental protocol. C2C12 cells in proliferative phase, in differentiation, and in postdifferentiation were treated with 5 mM CARN.

Proliferating cells, differentiating myocytes, and neomyotubes were treated with 5 mM CARN, the bioactive L-isomer of carnitine. This dose was chosen after a preliminary dose-response assay to establish the effective dose for the treatment (data not shown). In the control cells CARN was not added to medium.  Figure 1 explains experimental study design in each phase of the protocol, with cell confluence percentage and treatments start time and duration.

### 2.4. Growth Curve and Cell Viability Test

To study CARN role in C2C12 myoblast proliferation, we performed growth curve assay as described in [16]. Briefly, C2C12 myoblasts were plated in 60 mm × 15 mm culture dishes at 40% confluence and grown in GM with or without CARN and in DM. Medium was changed every 24 h and the experiment lasted until control cells achieved 70% of confluence (3 days). Every day, the cells were trypsinized, stained with trypan blue, and counted using a hemocytometer. The average values for each single day were used to plot a growth curve. Cell viability was calculated by dividing the nonstained viable cell count by the total cell count. In addition, morphological changes were examined daily by phase contrast microscopy.

### 2.5. Western Blot Analysis

Protein extracts, performed as described elsewhere [17], were obtained from cell cultures by using the following lysis buffer containing: 50 mM Tris/HCl, pH 7.4, 150 mM NaCl, 1% Triton X-100, 1 mM sodium orthovanadate (Na_3_VO_4_), 1 mM EDTA, 1 mM PMSF, 1 mg/mL aprotinin, 1 mg/mL leupeptin, and 1 mg/mL pepstatin.

Aliquots of 30 *μ*g supernatant proteins, quantified using Bradford method, were resolved on SDS-PAGE gel and transferred onto nitrocellulose membrane (Protran, Whatman Schleicher & Schuell). The membranes were incubated with specific primary antibodies and then with HRP conjugated anti-species-specific secondary antibodies. To confirm equal protein loading per sample, antibody anti-calnexin or anti-GAPDH was used. Quantitative measurement of immunoreactive bands intensities, visualized by an enhanced chemiluminescence method (Amersham Pharmacia Biotech, Piscataway, NJ, USA), was performed by densitometric analysis using the Scion Image software (Scion Corporation, Frederick, MD, USA). Data were then converted into fold-changes (FC) of the controls [18].

### 2.6. Immunofluorescence Analysis

C2C12 cells, fixed and permeabilized as described in [19], were blocked with PBS containing 1% bovine serum albumin. Slides or cells were then immunostained with specific antibodies rhodamine-conjugated and nuclei-revealed with DAPI staining. Slides were mounted with Moviol. Cells were observed using Nikon Eclipse 50I microscopy and images were captured using Nis-Elements D 4.00 software (Nikon Instruments Europe BV, Netherlands). Data were displayed and analyzed using Adobe Photoshop CS4. Live C2C12 cells were examined and images acquired by phase contrast microscopy using the same microscope and digital system described above.

### 2.7. Statistical Analysis

All experiments were performed three times. Data are presented as the mean ± SD. Statistical significances were assessed by *t*-test or Anova tests as appropriate. Results were considered significant when *P* ≤ 0.05.

## 3. Results

### 3.1. CARN Action during Myoblast Proliferation and Myocytes Commitment

**Figure 2:**
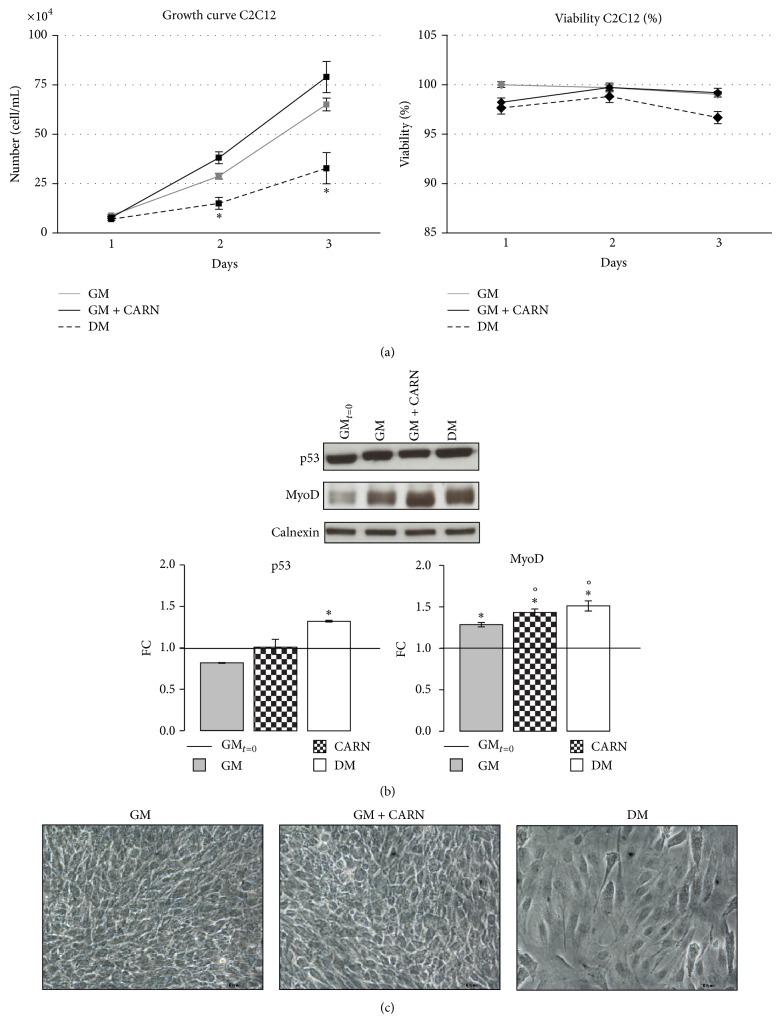
CARN action during C2C12 proliferation phase. (a) Growth curve: CARN did not significant modify C2C12 proliferative potential and did not induce cytotoxic effects. (b) CARN treatment does not modify p53 protein levels while it increases MyoD protein content after 3 days of treatment. (c) Phase contrast images reveal the morphological features of the cells in proliferation. Representative immunoblots of analyzed proteins are shown. Scale bar 200 *μ*m. Significance: (a) ^*^ *P* ≤ 0.05 DM versus GM and (b) p53 ^*^ *P* ≤ 0.05 DM versus GM_*t*=0_. MyoD: ^∙^ *P* ≤ 0.001 Anova test, ^*^ *P* ≤ 0.05 GM versus GM_*t*=0_, CARN versus GM_*t*=0_, DM versus GM_*t*=0_, °*P* ≤ 0.05 CARN versus GM, and DM versus GM.

We initially sought to determine the effect of exogenous CARN supplementation on myoblasts cell proliferation (Figure 2). Cells were cultured in three different media for three days: GM, GM supplemented with 5 mM CARN, and DM. CARN action on C2C12 proliferation capacity was studied assessing viability test and growth curve trend. DM exerted an inhibitory effect on C2C12 proliferative capacity, differently from CARN and GM superimposable effect (Figure 2(b)). Under the conditions investigated, cell viability was not negatively affected by the CARN treatment (Figure 2(a)). This result was confirmed by unstimulated p53 expression after 3 days of CARN supplementation (Figure 2(b)).

Also, while GM and CARN treated cells preserved their morphological characteristics at confluence, DM cells showed a morphology more similar to polarized cells (Figure 2(c)).

Because myogenic differentiation proceeds through irreversible cell cycle arrest of myoblasts [20], these data suggest that CARN is not still able at this stage to support the first step of differentiation process.

Skeletal muscle differentiation is governed by tight regulation of both activity and expression of a number of transcription factors, particularly the MRFs family members (MyoD, Myf5, Myogenin, and Myf6) [21]. Among these, MyoD and Myf5 are closely related to commitment process and early myogenic differentiation. Treatment of C2C12 myoblasts with CARN significantly increased MyoD protein level similarly to DM (FC: CARN 1.43 ± 0.04, FC: DM 1.51 ± 0.06; *P* ≤ 0.02 versus GM_*t*=0_, *P* ≤ 0.05 versus GM 24 h; GM_*t*=0_ versus GM, CARN, DM ^∙^ *P* ≤ 0.001 Anova test, Figure 2(b)).

**Figure 3:**
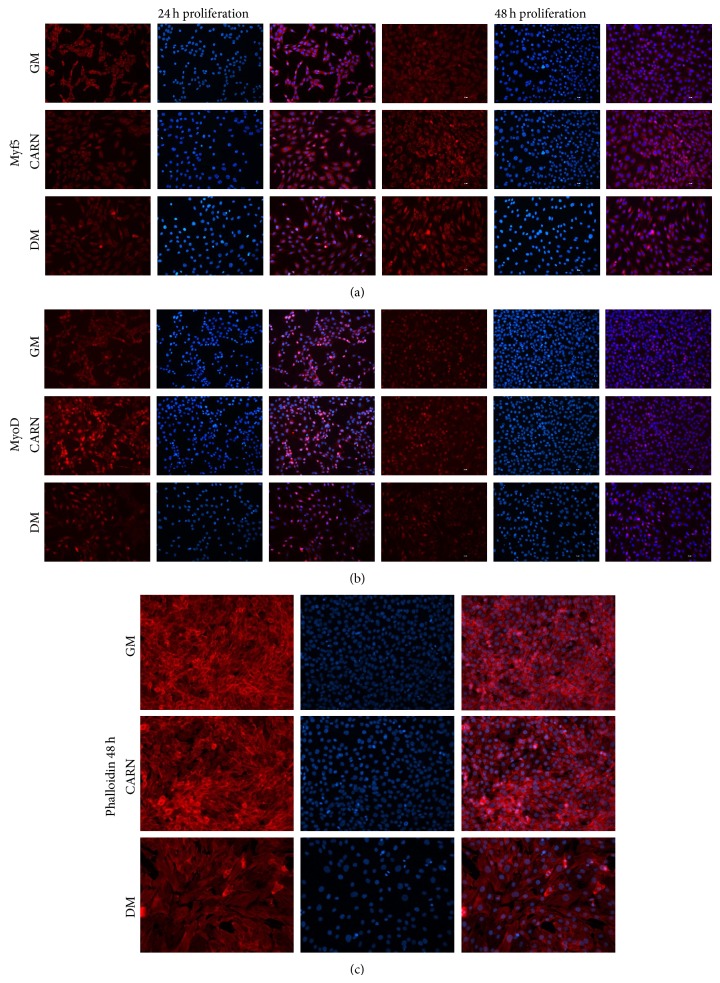
CARN action on C2C12 proliferating myoblasts study. (a), (b) Immunofluorescence analysis of early myogenic transcription factors Myf5 and MyoD during proliferative phase (24 and 48 h). CARN causes important cells morphological changes in respect to control: in particular, at 48 h, myoblasts lost their characteristic circular shape (GM) to achieve a new elongated morphology. (c) Phalloidin Immunofluorescence assay describes the important morphological changes in myoblasts treated with CARN. Scale bar 200 *μ*m.

As a complementary approach, we analyzed the morphology of CARN myoblasts by Immunofluorescence analysis performed using antibody against Myf5 and MyoD. Remarkably, CARN and DM myoblasts Immunofluorescence data were superimposable (Figures 3(a) and 3(b)). CARN stimulation caused important cell morphology changes in respect to GM. In particular, at 48 h, myoblasts lost the circular shape characterizing the GM cells to achieve an elongated morphology. This observation is further confirmed by Phalloidin Immunofluorescence assay (Figure 3(c)).

Together, these results suggest that CARN, although it does not arrest cell cycle, could enhance MRFs expression and then promote myoblasts commitment during proliferative phase.

### 3.2. CARN Action during Myoblast Differentiation: Enhancing Myotubes Formation

**Figure 4:**
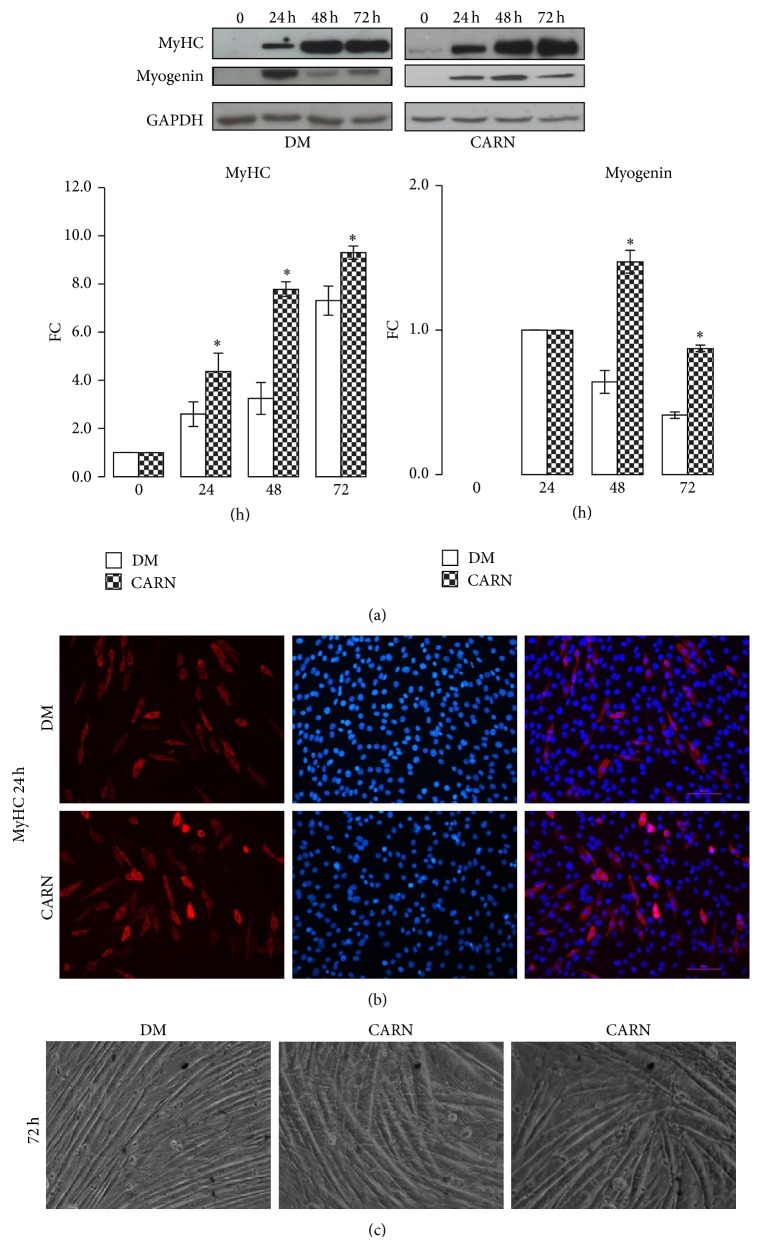
CARN action during myoblast differentiation: enhancing myotubes formation. (a) CARN enhances myotube formation rising MyHC and myogenin protein levels. (b) MyHC immunofluorescence assay revealed that CARN is able to enhance myotubes formation. (c) Phase contrast images at the end of differentiation show the important morphological changes and dimensional increment in neoformed myotubes after treatment with CARN. Representative immunoblots of analyzed proteins are shown. Scale bar 200 *μ*m. Significance: ^∙^ *P* ≤ 0.001 Anova test and ^*^ *P* ≤ 0.05 DM versus CARN at 24 h, 48 h, and 72 h.

We investigated CARN action during the different phases of muscle differentiation. To induce differentiation, C2C12 myoblasts were maintained in GM up to ~70% confluence (time 0) and then shifted in DM or DM supplemented with 5 mM CARN. Differentiating C2C12 cells were observed after 24, 48, and 72 hours (Figure 1). To analyze the possible effect of CARN on myogenesis progression, the protein levels of muscle-specific markers myogenin and MyHC were determined by Western blot analysis. As shown in Figure 4(a), myogenin and MyHC amount were increased by CARN presence (DM versus CARN ^∙^ *P* ≤ 0.001 Anova test, in particular FC myogenin 48 h: DM 0.64 ± 0.02, CARN 1.47 ± 0.08, *P* ≤ 0.02; myogenin 72 h: DM 0.41 ± 0.03, CARN 0.87 ± 0.02, *P* ≤ 0.02; FC MyHC 24 h: DM 2.60 ± 0.34, CARN 4.37 ± 0.75, *P* ≤ 0.05; MyHC 48 h: DM 3.25 ± 0.81, CARN 7.95 ± 0.02, *P* ≤ 0.01; MyHC 72 h: DM 7.31 ± 0.07, CARN 9.30 ± 0.28, *P* ≤ 0.005), indicating that CARN could promote myotubes formation. Moreover, we investigated whether CARN was able to modify morphological features of C2C12 cells after 24 h of differentiation, when myoblasts start to fuse in new myotubes. Immunofluorescence assay indicated that there is a higher myotubes number in CARN stimulated C2C12 group compared to control, confirming that CARN is able to enhance myotubes formation (Figure 4(b)). At the end of differentiation process, brightfield microscopy revealed that CARN myotubes exhibited increased diameter and length compared to control cells (Figure 4(c)).

### 3.3. CARN Action during Myoblast Differentiation: Signaling Pathway Activation

**Figure 5:**
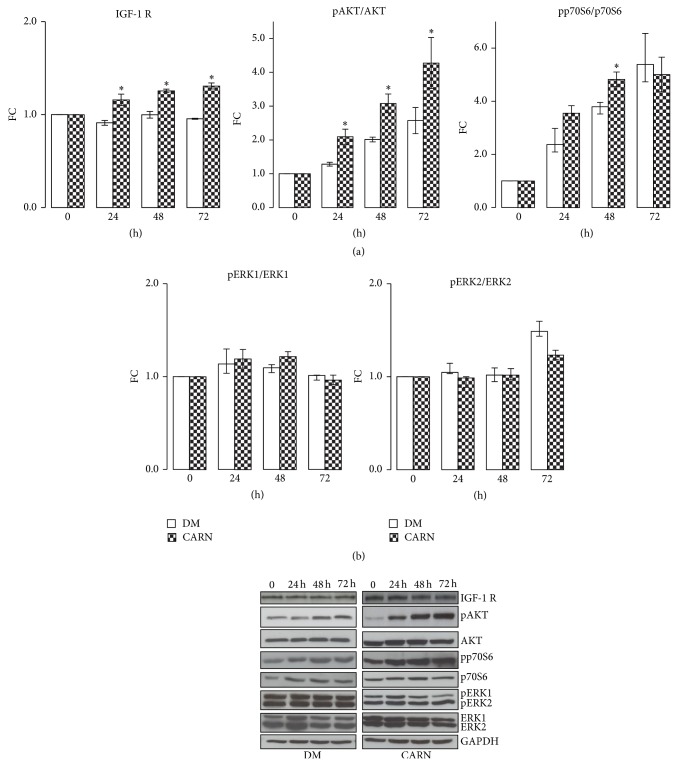
CARN action during myoblast differentiation: signaling pathway activation. (a) CARN stimuli significantly activate the IGF-1/AKT/p70S6 pathway. (b) CARN supplement did not have any effects on ERKs activation. Representative immunoblots of analyzed proteins are shown. Significance: ^∙^ *P* ≤ 0.001 Anova test and ^*^ *P* ≤ 0.05 DM versus CARN at 24, 48, or 72 h.

We investigated whether CARN was involved in skeletal differentiation, analyzing the main signaling pathways involved in the process: IGF-1/AKT/p70S6 and extracellular signal-regulated kinase 1/2 (ERKs). Skeletal muscle is a tissue highly responsive to IGF-1/AKT/p70S6. IGF-1 has been shown to be able to induce hypertrophy through either autocrine or paracrine mechanisms [22, 23]. AKT action has been linked to skeletal muscle development, regeneration, and hypertrophy through several pathways that culminate in stimulation of protein synthesis, inhibition of atrophy, and prevention of cell death [24]. p70S6K, a positive regulator of protein translation, is an AKT target [25]. ERKs can be activated by a variety of growth factors/mitogens. ERKs activation has been shown to be crucial for growth factor-induced myoblasts proliferation, fusion, and hence myoblasts differentiation [26]. As shown in Figure 5(a), CARN significantly increased the amount of IGF-1 receptor (DM versus CARN ^∙^ *P* ≤ 0.001 Anova test, in particular 24 h: DM 0.91 ± 0.03, CARN 1.16 ± 0.06, *P* ≤ 0.02; 48 h: DM 1 ± 0.04, CARN 1.26 ± 0.02, *P* ≤ 0.01; 72 h: DM 0.95 ± 0.01, CARN 1.31 ± 0.03, *P* ≤ 0.002) and promoted AKT (^∙^ *P* ≤ 0.001 Anova test, in particular 24 h: DM 1.28 ± 0.06, CARN 2.09 ± 0.23, *P* ≤ 0.02; 48 h: DM 2.01 ± 0.07, CARN 3.08 ± 0.28, *P* ≤ 0.02; 72 h: DM 2.57 ± 0.39, CARN 4.28 ± 0.75, *P* ≤ 0.05) and p70S6 kinase phosphorylation (24 h: DM 2.37 ± 0.61, CARN 3.55 ± 0.28, *P* ≤ 0.05; 48 h: DM 3.79 ± 0.14, CARN 4.83 ± 0.27, *P* ≤ 0.02). In contrast, CARN did not show any effects on ERKs activation (Figure 5(b)). These results demonstrate that CARN enhances myogenic differentiation acting on IGF-1/AKT/p70S6 pathway.

### 3.4. CARN Action during Myoblast Differentiation: Antioxidant Action

**Figure 6:**
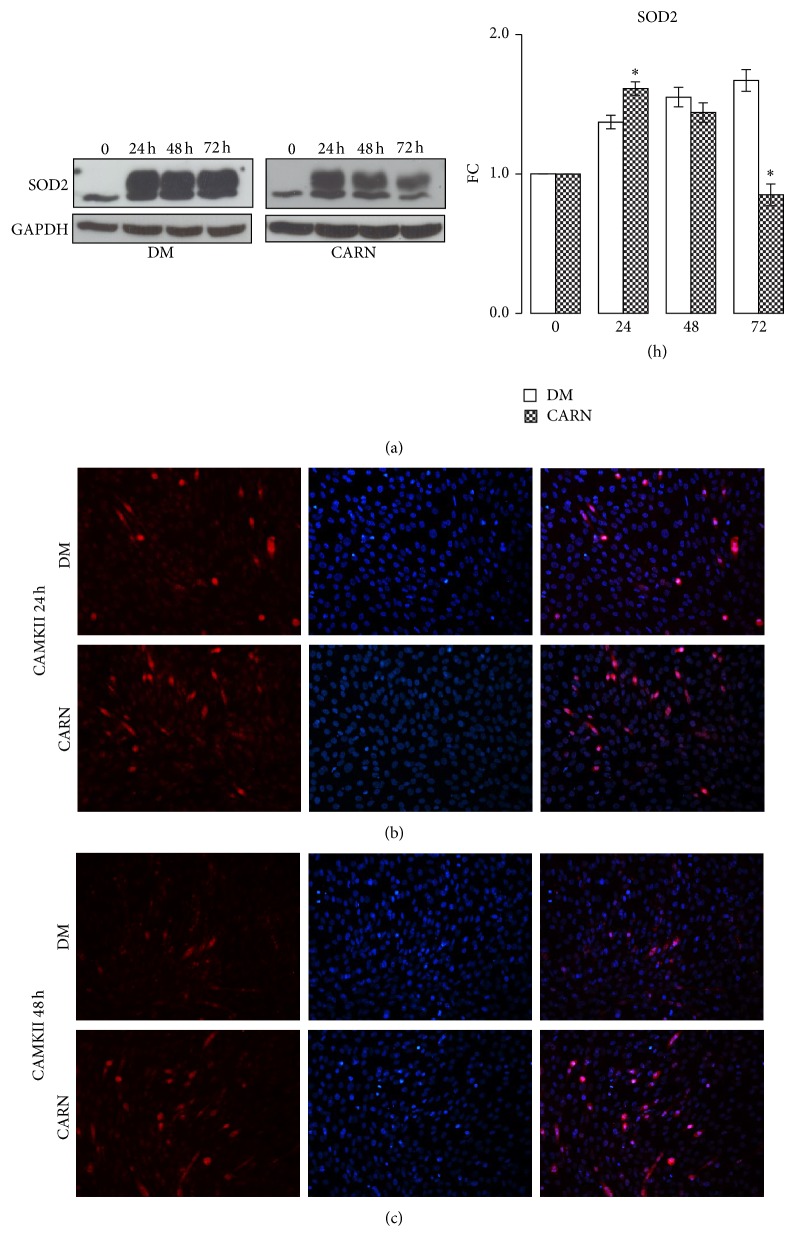
CARN action during myoblast differentiation: antioxidant action. (a) CARN positive modulate SOD2 protein synthesis, key enzyme in oxidative stress response. (b), (c) Immunofluorescence staining results show that the CAMKII expression in CARN nascent myotubes is higher than that in control group. Representative immunoblots of analyzed proteins are shown. Scale bar 200 *μ*m. Significance: ^∙^ *P* ≤ 0.001 Anova test and ^*^ *P* ≤ 0.05 DM versus CARN at 24, 48, or 72 h.

SOD2 is a critical mitochondrial antioxidant defense against superoxide produced by respiration [27]. As shown in Figure 6(a), 5 mM CARN increased SOD2 protein level during early differentiation phase (^∙^ *P* ≤ 0.001 Anova test, in particular 24 h: DM 1.37 ± 0.04, CARN 1.62 ± 0.05, *P* ≤ 0.04). In addition, the multifunctional Ca^2+^ and calmodulin-dependent protein kinase II (CaMKII) is recognized to play a crucial role for normal mitochondrial function and, in particular, in antioxidative metabolism [28]. Immunofluorescence staining results (Figures 6(b) and 6(c)) showed that the CAMKII expression in CARN stimulated neomyotubes was higher than in control group (24 h–48 h), suggesting that CARN could ameliorate not only myotubes formation but also antioxidative mitochondrial pathways.

### 3.5. CARN Action on Neoformed Myotubes

**Figure 7:**
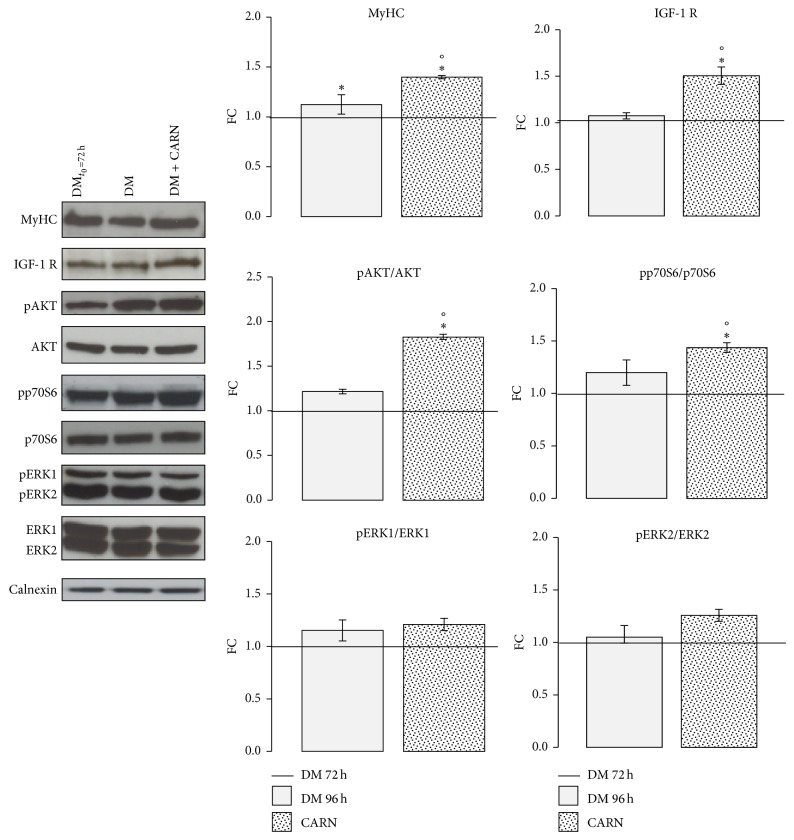
CARN action on neoformed myotubes. To study CARN action on C2C12 myotubes, neoformed myotubes (after 72 h to differentiation induction) were stimulated for 24 h with 5 mM CARN. Graphs of Western blot analysis during postdifferentiation phase show the significant increment of MyHC protein level in neoformed myotubes treated with CARN. In addition, CARN increases IGF-1 receptor and activates AKT and pp70S6 protein kinases. Representative immunoblots of analyzed proteins are shown. Significance: ^∙^ *P* ≤ 0.001 Anova test and ^*^ *P* ≤ 0.05 DM versus DM_*t*=72 h_, CARN versus DM_*t*=72 h_, and °*P* ≤ 0.05 CARN versus DM.

To study CARN action on C2C12 myotubes, neoformed myotubes (after 72 h of differentiation) were stimulated with 5 mM CARN for 24 h. Western blot analysis confirmed the evidence observed in differentiation phase: CARN increased the MyHC protein content (^∙^ *P* ≤ 0.001 Anova test, in particular DM96h 1.12 ± 0.1, CARN 1.40 ± 0.01, *P* ≤ 0.05 versus DM96h), activating IGF-1/AKT/p70S6 signaling pathway (^∙^ *P* ≤ 0.001 Anova test, in particular IGF-1 R: DM96h 1.08 ± 0.03, CARN 1.51 ± 0.09, *P* ≤ 0.01 versus DM96h; pAKT/AKT: DM96h 1.22 ± 0.03, CARN 1.03 ± 0.03, *P* ≤ 0.001 versus DM96h; pp70S6/p70S6: DM96h 1.20 ± 0.12, CARN 1.44 ± 0.05, *P* ≤ 0.05 versus DM96h) and did not influence ERKs pathways (Figure 7).

## 4. Discussion

In the last decades, numerous studies investigated the positive CARN supplement action on physical performance shedding new light on the importance of CARN as a regulator of skeletal muscle fuel selection and physiological function (long-chain fatty acids transport), with CARN role not only as ergogenic aid but also as pharmacological treatment in pathological conditions characterized by muscle damage [7, 8, 29].

However, to date, no research investigated whether CARN could positively affect muscle differentiation process.

Our work is the first study to show that increasing skeletal muscle CARN content in murine immortalized C2C12 cell line can modulate differentiation process regulating protein synthesis signaling and oxidative stress response.

The activation of the myogenic regulatory factors Myf5 and MyoD is required to stop satellite cells progression and to turn on myoblast commitment towards early muscle cell differentiation [20–22]. In our* in vitro* study, CARN promotes the expression of these factors (Figures 2 and 3) implying its ability to strengthen the satellite cells commitment.

Moreover, CARN enhances the myotubes differentiation stimulating the expression of the late myogenic regulatory factor myogenin and skeletal muscle protein MyHC (Figure 4), modulating IGF-1/AKT/p70S6 signaling pathway (Figures 4 and 5). Moreover, CARN enhances the myotubes differentiation stimulating the expression of the late myogenic regulatory factor myogenin and skeletal muscle protein MyHC (Figure 4). This and modulating IGF-1/AKT/p70S6 signaling pathway (Figures 4 and 5). The same regulatory pathway is still activated by CARN in the neoformed myotubes (Figure 7). Our data confirm recent* in vivo* evidence showing that CARN supplementation increases plasma IGF-1 concentrations and its signaling pathway [30–32]. Moreover, a significant work, which analyzed the CARN treatment effects on microRNA expression profile in rat skeletal muscle, suggests that the IGF-1 raise is mediated at the level of microRNA [33].

Interestingly, several studies have indicated that IGF-1 not only influences muscle hypertrophy but also inhibits muscle protein degradation, responsible of skeletal muscle atrophy [23, 34, 35]. This effect is induced by AKT signaling pathway activation, which inhibits the expression of the proteins (in particular MuRF1 and atrogin-1) involved in ubiquitin-proteasome system (UPS), a highly regulated mechanism of intracellular protein degradation [24, 35, 36]. Corroborating this evidence, several research works, in animal models, described how CARN supplementation leads to a downregulation of genes of the ubiquitin proteasome system (UPS), modulating the release of inhibitors of the UPS such as IGF-1 [37–39].

Our results not only confirm the stimulatory effect of CARN on IGF-1/AKT/p70S6 pathway but also show that this effect continues throughout the process of differentiation. They also show the ability of the CARN to strengthen the process of myoblasts differentiation. Supported by these data, we allow recognizing CARN ability to exert beneficial effects on muscle regeneration, particularly under conditions associated with an increased activity of the UPS, like in cancer-related cachexia or sarcopenia state [40, 41].

Sarcopenia, the loss of skeletal muscle mass and function that occurs with aging, is an accompanying loss in strength, aerobic capacity, and metabolic rate that contribute to the reduced function and quality of life [37–41]. The current first-line therapy for preventing and treating sarcopenia is resistance exercise [6, 41, 42]. Based on our data, since CARN promotes muscle commitment, differentiation, and IGF-1/AKT/p70S6 pathway activation, it might represent an attractive new drug for the prevention and treatment of sarcopenia. Furthermore, in our previously published work, we showed that in a particular category of patients with lipodystrophy, L-acetyl-carnitine supplementation may be able to ameliorate the body composition [43]. In addition, CARN seems to be able not only to modify the body composition but also to promote muscle fiber transition, how demonstrated in rat obese model [44].

In addition, we observed that during differentiation CARN increased the key proteins involved in antioxidant process (Figure 6), in line with some* in vivo* and* in vitro* studies on CARN antioxidant and antiradical activities [45, 46]. This antioxidative action of CARN strengthens its position as a promising candidate for the prevention and treatment of associated muscle injury conditions. In fact, CARN shows a dual role in countering the development of muscle wasting: from a side by opposing the formation of ROS, potent UPS inducers, and on the other enhancing IGF-1/AKT/p70S6 pathway, potent inhibitor of UPS.

Typically, there is an inverse relationship between efficient muscle fatty acid *β*-oxidation and reactive oxygen species (ROS) production [46, 47]. Previous studies have concluded that increased oxidative stress developed in response to lipid overload causes muscle insulin resistance. In fact, patients with insulin resistance and/or type 2 diabetes mellitus often have impaired skeletal muscle oxidative capacity and inefficient or incomplete fatty acid *β*-oxidation [48].

It is important to emphasize that our data allow us to hypothesize that, in insulin resistance conditions, CARN supplementation might prevent both incomplete fatty acid *β*-oxidation and oxidative stress process, counteracting the occurrence of muscle insulin resistance conditions.

In summary, considering its importance in muscle bioenergetics and its antioxidant potential, CARN supplementation may be considered an aid in condition of CARN deficiency and in skeletal muscle diseases. Despite this potential, further researches are needed to conclusively elucidate the mechanisms underlying its protective effects and to verify their safety and efficacy in treatment of a number of different muscle diseases.
